# Postmortem minimally invasive tissue sampling in communities: exploring perceptions of families, funeral workers, religious and community leaders and healthcare providers from Pakistan

**DOI:** 10.1186/s12913-023-10438-2

**Published:** 2023-12-13

**Authors:** Nazia Ahsan, Fauzia Aman Malik, Waliyah Mughis, Rawshan Jabeen, Shaheen Mehboob, Raheel Allana, Syeda Quratulain, Saima Jamal, Christina R. Paganelli, Norman Goco, Lindsay Parlberg, Saad B. Omer, Abdul Momin Kazi

**Affiliations:** 1 Department of Paediatrics and Child Health, Aga Khan University, Karachi, Pakistan; 2 Yale School of Public Health, Yale University, New Haven, Connecticut, USA; 3 Social, Statistical and Environmental Sciences, RTI International, Research Triangle Park, Seattle Washington, NC USA; 4 Yale Institute for Global Health, Yale University, New Haven, Connecticut, USA

**Keywords:** Minimally invasive tissue sampling, Community perceptions, Community-based participatory qualitative research, Child heath, Asia

## Abstract

**Background:**

Minimally invasive tissue sampling (MITS) has increasingly been used to improve the diagnosis of disease and identification of the cause of death, particularly in underserved areas. However, there are multiple barriers to accessing those who die within the community, our study aimed to explore the perceptions and insights of community members and healthcare providers regarding the feasibility of implementing MITS in community settings.

**Methods:**

A qualitative exploratory study was conducted. A total of twenty one in-depth interviews were conducted with deceased infants’ parents, elders of the family, religious leaders, community leaders, and funeral workers. Focus group discussions were conducted with health care providers (n = 14) in two peri-urban slum areas of Karachi, Pakistan. The duration of this study was from August to October 2020. Data was analyzed using thematic analysis and was coded and merged into categories forming eight major themes.

**Results:**

In general, participants viewed minimally invasive tissue sampling (MITS) as beneficial for improving child health, though some had concerns about disrespecting the deceased during sample collection. Misinformation, fear of needles, and medical procedures were major barriers to MITS implementation. To enhance acceptance, community and religious leaders suggested using religious rulings, obtaining parental consent, ensuring confidentiality, and increasing efforts of community engagement. Community healthcare providers, along with funeral workers, recommended providing community members with grief counseling to increase study participation. Besides concerns about sampling interfering with respect for the decease, community members also raised concerns about misinformation. Further, participants provided feedback on the design and appearance of the mobile van used to collect MITS samples from children under the age of five.

**Conclusion:**

This study is critical for understanding the challenges associated with implementation of community-based MITS sampling in Pakistan. Integrating the use of a mobile van for sample collection, grief counseling along with community engagement sessions and advocacy will help address community-based misinformation and develop community trust.

**Supplementary Information:**

The online version contains supplementary material available at 10.1186/s12913-023-10438-2.

## Background

Childhood mortality remains a major issue worldwide, with approximately 5 million children under the age of five dying in 2020 [1]. To reduce neonatal, infant, and child mortality rates, it is crucial to identify the causes of these deaths (CoD), many of which can be prevented through affordable and basic quality interventions [2, 3]. In low middle income countries (LMICs), deaths occurring within the community are frequently associated with respiratory illnesses or sudden infant death syndrome based on verbal autopsy data [4]. However, methods such as verbal autopsies are not always effective in accurately determining the cause of death [5, 6]. An alternative approach is Minimally Invasive Tissue Sampling (MITS), a less-invasive alternative to full autopsy which involves the targeted sampling of specific organs through small incisions or needle biopsies. These samples can then be analyzed in the laboratory to determine the cause of death with a high degree of accuracy [7]. Previous studies have compared minimally invasive tissue sampling with the gold standard full-autopsy and reported relevant diagnostic concordance with complete diagnostic autopsy [6].  . A recent study by Tanko et al. reported MITS to be a valuable and reliable diagnostic procedure for the identification of CoD in children, stillbirth, and neonates which can be used to contribute vital mortality statistics if complete diagnostic autopsy is unavailable [8]. Importantly, MITS allows for the collection of tissue samples with minimal invasiveness, reducing disruption to the body of the deceased while enabling healthcare professionals to obtain accurate and reliable information [9]. One of the key advantages of MITS is its potential to overcome cultural or religious barriers that may limit the acceptance or feasibility of full autopsy in certain communities [10]. It also provides a culturally sensitive alternative for families who may wish to respect the body of the deceased while still identifying a CoD [11].

In high-income countries, MITS has proven to be a valuable tool for identifying CoD related to infectious diseases like tuberculosis, HIV/AIDS, and malaria [12–14]. However, implementation of MITS in LMICs has been limited due to several factors, including inadequate access to pathology services, lack of personnel trained in MITS, and limited availability of essential equipment [15]. In Pakistan, the lack of regular processing of cause-of-death statistics by the National Statistics Bureau poses an additional challenge [16]. This lack of reliable data can hinder efforts to understand the true burden of disease in a population, identify trends in mortality, and develop evidence-based policies to improve health outcomes [15].

In our previous Respiratory Syncytial Virus (RSV) mortality surveillance study, we explored community perceptions on the acceptability and feasibility of nasal swab and MITS sample collection at four health demographic surveillance system sites. Qualitative studies have highlighted that, despite the potential advantages of MITS [17], such sampling techniques may not be suitable for countries like Pakistan due to cultural and religious beliefs, and differences in healthcare system infrastructure. Insufficient evidence identified that MITS was acceptable in Pakistan e.g., understanding the attitudes and opinions of healthcare workers towards MITS was the goal of a qualitative study carried out at the Hospital Karachi, Pakistan [18]. In the RSV study, we sought to address these cultural challenges by obtaining religious approvals of the sampling from religious scholars and sharing these with the community through continuous engagement activities [19]. A qualitative study conducted Pakistan reported that MITS was seen as a practical method for determining cause of death in stillbirths and newborns [20] and also described elements that might make implementation of MITS easier in Pakistan and other similar contexts [20, 21]. However, no evidence was reported on how best to conduct MITS at the community level in Pakistan. To effectively conduct community-based MITS, it is crucial to consider the perspectives and attitudes of healthcare providers, religious leaders, and community members, as they can play a significant role in influencing the acceptance and uptake of the procedure by their communities. Furthermore, exploring community perceptions of MITS can help identify potential obstacles to acceptance, and develop strategies to overcome these obstacles. In this study, we explored community perception’s of MITS and the acceptability of conducting community-based MITS among families, funeral workers, healthcare providers, religious and community leaders. Additionally, our study introduced a novel mobile van designed to enable the collection of MITS samples in the community: equipped with the necessary tools and supplies, the van allows researchers to conduct MITS procedures on site, and for the efficient and safe transportation of the samples to research facilities. The introduction of a van is a significant step towards improving the accessibility and acceptability of MITS in community settings, particularly in areas with limited availability of laboratory/medical testing services, skilled staff, and necessary equipment.

## Methods

### Study design and setting

The study employed a qualitative exploratory design and focused on a formative phase in anticipation of MITS implementation. The study was conducted in two peri-urban settlements of Karachi, Pakistan: Bhains Colony (BHC) and Ali Akbar Shah Goth (AAG). The Aga Khan University has a Health Demographic Surveillance System that records newborn morbidity and mortality in both communities. These settlements are situated in the coastal area of Karachi, where people of various ethnicities reside together. The total population was more than 82,808 in Ali Akbar Shah Goth and 81,792 in Bhains Colony. Most men work as fishermen, laborers, or daily wage earners, and speak various languages, including Sindhi, Punjabi, Pashto, Bengali, and Urdu [22]. These communities were predominantly Muslim, in which the majority of death processes end with burial. The researchers developed semi-structured guides for data collection based on knowledge gained from previous mortality surveillance studies in these areas, literature reviews, and research aims, which identified key topics for discussion during the study [23, 24] (See Tables 1 and supplementary I). Data was collected through in-depth interviews and focus group discussions from August to October 2020. Ethical approval was granted by Aga Khan University Ethical Review Committee (AKU-ERC NO: 2021-3675-15525) before initiating this study.

**Table 1:** Topic checklist for the Focus group discussion and In-depth interviews

S.No.	Topics explored during discussion with study participants
1.	Perceived benefits and challenges in knowing the cause of death for children
2.	Perceptions and barriers of performing MITS in a sample collection van in community
3.	Explore ways of receiving death alerts for sample collection
4.	Beliefs and concerns of community that may conflict with MITS acceptability
5.	Perceptions of stakeholders to advocate MITS sampling in the community that would be socially and culturally accepted
6.	How the community perceives performing MITS procedure in a sample collection van

Study inclusion criteria required study participants to be a resident of either Bhains Colony (BHC) or Ali Akbar Shah (AAG), the peri urban areas of Karachi. Community members who refused to provide consent at the time of interviews were excluded from the study.

### Study participants

Twenty-one in-depth interviews (IDIs) were conducted with study participants including the parents of deceased infants, community elders, religious leaders, funeral workers (bathers, burial goods vendors, and graveyard undertakers) and community healthcare providers from AAG and BHC sites. Funeral workers were included in the study because bathers, shroud sellers, and undertakers are the first point of contact for families arranging funerals: for the purposes of MITS sample collection, these community members often notify the research team of child deaths in the community. In addition to IDIs, two focus group discussions were conducted which included fourteen healthcare providers with at least 1 to 25 years of experience within the same sites. The healthcare providers included doctors, lady health workers (LHWs), vaccinators, union council communication support officers (UCCSO-Polio), local community health workers (CHWs), and their supervisors. These participants belonged to various ethnicities, and while Punjabi, Hindko, and Sindhi were the most spoken languages, participants understood and were comfortable communicating in the Urdu language. Table 2 provides further details on this.

### Data collection process and management

**Fig. 1:**
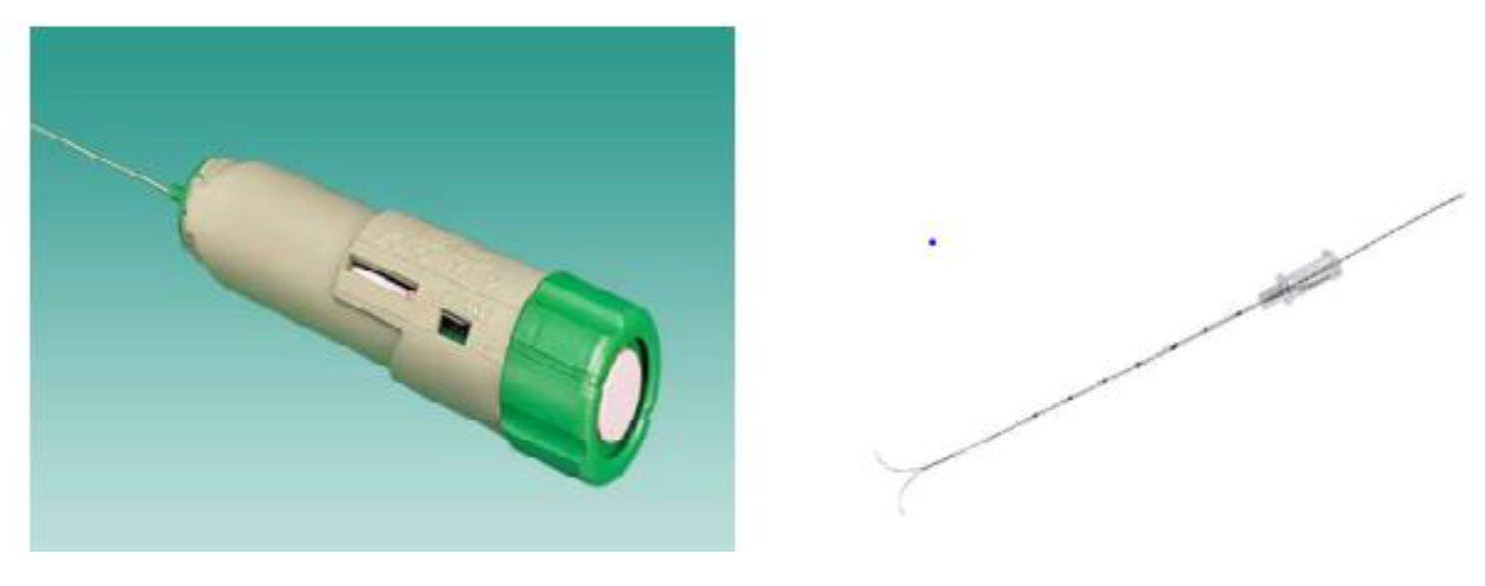
MITS needle shown to the participants during the study FGDs and IDIs

Interview guides for both focus group discussions (FGDs) and in-depth interviews (IDIs) were developed, pre-tested, and translated into local language (e.g., Urdu). Additionally, participants were shown pictorial aids of the MITS needles and sampling techniques to improve their understanding of the MITS procedure (Fig. 1). Individual participants signed a consent form after they agreed to participate. All participants’ response data was digitally recorded and transcribed verbatim locally before being translated into English. We systematically explored the transcripts to identify recurring themes, insights, and patterns in our qualitative data, which included twenty one in-depth interviews and two focus group discussions (FGDs). Concurrently, we compared emergent information between participants to ensure that all perspectives were captured. Iterative data collection and analysis were carried out until no new significant information emerged from the in-depth interviews and focus groups, similar to methods applied in other studies [20, 25]. This meticulous procedure ensured that our research captured the range and depth of participant experiences, attaining saturation and boosting the robustness of our study’s findings [26]. The point of saturation was reached in our qualitative interviews when we noticed that we were consistently hearing the same themes and responses from participants, indicating that we had gathered a comprehensive understanding of the themes. All interview audio recordings were labelled with unique ID numbers to prevent them from being lost or mixed. The same unique ID was used to label the respective transcriptions and written field notes. All files were kept in a password-protected computer and saved on an encrypted cloud server that is only accessible by the research team. We used Lincoln and Guba’s criteria to strengthen the quality of search and trustworthiness [10] and in order to ensure that researchers followed rigorous standards for data collection and analysis to produce accurate and reliable results as well as valid and credible findings [27].

### Analysis

All data collected was assessed using thematic analysis, in which transcripts were read several times by two independent researchers to develop a comprehensive interpretation. The translated transcription data were coded, compared, and refined to generate emergent themes. The transcribed text was divided into ‘meaningful units’, and later shortened and labeled with a code. The translated transcription data were coded, compared, contrasted, and refined to generate emergent themes e.g., data were thematically analyzed by two separate researchers, who created codes and categories. After conducting an independent read of the data, the two researchers collaborated to reduce researcher bias and address any disagreements. Several additional measures were taken to ensure complete and unbiased collection of data. First, two secondary coders (Qualitative researchers) who were not involved in the larger study process reread transcripts from FGDs and IDIs. To find emerging themes, three senior researchers (a social scientist, a psychologist, and an anthropologist) independently analyzed the transcript to generate themes and categories to further resolve discrepancies to reduce bias. Finally, the opinions of different study participants and investigators were verified using different data sources to ensure credibility of the study [27].

## Results

A total of twenty one in-depth interviews and two focus group discussions were conducted with parents, community elders, religious leaders, funeral workers (bathers, burial goods vendors, and graveyard undertakers) and community healthcare providers to explore community insights on the MITS procedure and its use at community level.

Table 2 represents the number of participants involved in in-depth interviews and focus group discussions.

**Table 2:** Detail of Stakeholders participating in FGDs and IDIs.

In-depth Interview		No. of participants
1	Elders (Deceased grand parents and community elders)	4
2	Parents	3
3	Religious leaders/Community leaders	6
4	Funeral workers	8
**Focus group discussion**		**No. of participants**
5	Healthcare providers	14 (7 participants in each FGDs)

### Thematic analysis

The perceptions of key stakeholders were categorized under eight major themes which are described below in detail.

### 1. Drivers of community acceptance and understanding of MITS

During interviews, elderly members of household reported an acceptance of the MITS procedure as they believed that a deceased child never returns to life and viewed MITS as comparable to other types of postmortem examinations, such as the complete diagnostic autopsy:

*“MITS samples can be obtained from deceased person who cannot come back to life in case of an accident or homicide. It is customary for doctors to perform postmortem tests in such situations. If the parents provide consent, then one can obtain a sample, and it is suggested for all to allow it.” (IDI: Grandfather of deceased infant - Ali Akbar Shah Site)*.

The parents of deceased infants also shared that they were willing to consider MITS testing options because they believed it was important to know the cause of their child’s death:

*“We do not know about community, but we are parents, and we do not allow it. If it is necessary you can do, you can do because to know the reason of death.” (IDI: Parent of deceased child - Ali Akbar Shah Site)*.

Respondents felt that implementation of MITS could provide comfort to parents who had lost a child by providing a cause of death to mitigate feelings of self-blame and guilt. Moreover, parents believed that MITS was a truly less invasive, and the results of MITS sampling would provide consolation to grieving families:

*“Parents will know the cause of death of the child, so they will be comforted. Otherwise, there is no consolation. The soul is tormented all the time. They think it is our fault.” (IDI: Parent of deceased child - Bhains Colony Site)*.

Most healthcare providers also perceived MITS as beneficial for improving child health, and expressed an understanding that MITS could generate new information that might improve medical outcomes for newborns and mothers who have suffered repeated neonatal deaths and stillbirths.

the community leaders highlighted high infant mortality rates in Pakistan, and expressed that these issues remained unresolved due to the unknown causes of death.

They also expressed that the research team, along with public health experts, should explore these CoDs and find solutions using the MITS procedure:

*“MITS’ benefits for community to get a sample; you can get this. In Pakistan, we have a big ratio of infant death, and we do not know the reason. If you are doing research and with this research, you will introduce the treatment of the disease, then it should be necessary.” (IDI: Community Leader - Ali Akbar Shah Site)*.

Community members also comprehended the importance of MITS for the prevention and management of disease spread. Particularly, a graveyard undertaker suggested that the residents of the Bhains Colony site might be willing to accept the use of MITS during a disease outbreak:

*“The community members will agree now a days (during pandemic situation) as it is an outbreak of COVID-19.” (IDI: Graveyard undertaker – Ali Akbar Shah Site)*.

*“MITS is beneficial for the community.” (FGD: Healthcare provider of Ali Akbar Shah Site)*.

Additional facilitators of acceptance identified in the study included parental consent, a key factor highlighted by both community leaders and healthcare providers; as well as the hopes for a new vaccine developed to save neonates and children:

*“This is a good thing that after the test, a vaccine of the disease can be developed and made available. This is particularly beneficial for diagnosing the disease in children” (FGD: Healthcare provider of Ali Akbar Shah Site)*.

### 2. Religious perceptive on MITS acceptance

People with low literacy levels were less inclined to accept MITS due to religious rulings, as expressed by a healthcare provider from Bhains Colony Site:

*“Illiterate people don’t believe in Fatwa because they are not able to read.” (FGD: Healthcare provider of Bhains Colony Site)*.

The shroud sellers shared that everything for the betterment of humanity is allowed in Islamic Sharia. Similarly, both healthcare providers and community leaders emphasized that religious leaders and their rulings “Fatwa” (a legal opinion ruling by an Islamic religious leader) would play a major influence on the community’s acceptance of MITS sampling within the community. Also, healthcare provider shared their concern that the people might not be able to read Fatwa as literacy is low.

*“If you have a religious leader with you, people will listen to him.” (FGD: Healthcare provider of Ali Akbar Shah Site)*.

*“There is no objection in the postmortem. There is no problem in getting a sample for the betterment of our children’s future. If sharia allows us to do it, you can get a sample.” (IDI: Shroud Sellers - Ali Akbar Shah Site).*

The Muslim religious leaders were also accepting of MITS, in keeping with a few major justifications: they perceived MITS sampling similar as postmortem for the decease, in line with Fatwas, and as part of charitable good deeds for human beings.

*“This is same as postmortem and sampling is also permissible as its for betterment of people as said “Whoever saves a life, it is as if they have saved all of humanity” Quran, the holy book of Islam, in Surah Al-Ma’idah (5:32). (IDI: Religious Leader – Ali Akbar Shah Site)*

*It is an ongoing charity as per Muslims religious belief” (IDI: Religious Leader - Bhains Colony Site)*.

### 3. Healthcare providers perceptions about MITS procedure acceptance

In focus group discussions, healthcare workers suggested that it was important for parents of the deceased to be approached for MITS participation before the larger family gathered for the death ritual to prevent interference by external factors:

*“If you will contact them earlier before people gathered at their place, they will agree to give you the sample.” (FGD: Healthcare provider of Bhains Colony Site)*.

Additionally, healthcare workers made recommendations in relation to building community trust and establishing appropriate clinical practices during sampling.

#### Building a trustworthy relationship within the community

Participants suggested that community-based or reliable and familiar medical physicians were appropriate ambassadors to build trust and obtain consent within the community. The community healthcare workers also suggested maintaining confidentiality while disclosing the findings of MITS to parents:

*“The report should not be handed over to anyone else. Physicians go themself and give it in the hands of mother or father.” (FDG: Healthcare provider of Bhains Colony Site)*.

Because the community has a high level of trust in healthcare providers - such as doctors and healthcare workers - when it comes to any diagnostic procedure, healthcare providers also recommended that a physician be the one to deliver findings to increase parental trust:

*“In the hospital, there are doctors and staff who are treating their child and people trust the doctors.” (FGD: Healthcare provider of Bhains Colony Site)*.

Finally, healthcare workers emphasized the need for advocacy and education within the community: they felt that the MITS procedure cannot be performed without providing both the community with information about sampling and the project at large, and the family with additional counseling.

#### MITS procedure under good clinical practice

According to healthcare providers, the common factors in underserved communities were disapproving attitudes towards the MITS procedure and being afraid of medical centers:

*“People will think positive and negative, and the negative thoughts will affect sample collection and people will be scared to come to us in health clinics.” (FGD: Community Healthcare worker of Ali Akbar Shah Site)*

Healthcare providers emphasized the importance of providing high quality laboratory procedures, such as sterilization for sample collection and the use of new kits for every sample, even in a mobile sampling van. One of the reasons healthcare workers demanded standard procedure was to minimize the risk of infection of other family members:

*“Separate sampling kits should be used for deceased sampling.” (FGD: Healthcare provider of Bhains Colony Site)*.

*“You need to communicate to people that van is free from germs and pollution and any germs absent in the body will not be reported.” (FGD: Healthcare provider of Ali Akbar Shah Site)*.

Similarly, the community leaders highlighted the need for awareness sessions and informing parents about the purpose of MITS and advantages it offers to the community.

*“There is an increased likelihood that parents and community members will consent to your team collecting a sample in the instance of a death if you share information about MITS with them and offer counseling services”.” (FGD: Healthcare provider of Bhains Colony Site)*.

### 4. Role of community engagement in acceptance of MITS

Healthcare providers shared that concerns they had previously during the Respiratory Syncytial Virus (RSV) project were resolved through advocacy. They emphasized the importance of advocacy and community engagement in resolving community concerns about MITS procedures, in the same way that these techniques were used in previous research studies:

It is possible that some community members may disseminate wrong or incomplete information about the MITS samples, but it is important to remember that the aforementioned RSV project previously addressed issues of a similar nature. These rumors may get the community’s attention, but it is crucial to remember that the prior initiative successfully dealt with analogous challenges while obtaining samples from community members who had passed away.

*“Some members of the community may spread rumors about the MITS samples, but similar concerns were addressed in a previous Respiratory Syncytial Virus (RSV) project where samples were collected from deceased individuals in the community.” (IDI: Community Leader – Ali Akbar Shah Site)*.

By providing regular community outreach advocacy to parents and community members, healthcare workers felt that the research team might make MITS more acceptable to the community:

*“By providing regular information on MITS and counseling to the parents and community members, they would be more likely to allow your team to collect a sample in the event of a death.” (IDI: Community Leader – Ali Akbar Shah Site)*.

### 5. Community perceptions regarding MITS sample collection van

In general, healthcare providers were happy with the idea of using a specialized van to conduct MITS sample collection within the community:

*“You can have sample in the van there is no problem to have sample in your sampling van.” (IDI: Mother of deceased child - Bhains Colony Site)*

They suggested that the mobile van should have an ambulance or healthcare logo (such as a plus sign) and be parked on the roadside:

*There should be a plus sign on the van, so people know that it is a lab van.” (FGD: Healthcare provider of Ali Akbar Shah Site)*.

The essential features of the mobile sample collection van described by community leaders and elders were measures to control infections and maintain hygiene, a hand washing area, air-conditioning, oxygen supply, emergency management equipment, and ambulance-like markings. Respondents also mentioned how crucial it would be to have specific facilities and equipment for cleaning in the vehicle. They emphasized the requirement for each component to be properly located within the van, as well as the need for water and soap to be easily available.

*“All facilities should be there such as air conditioning, emergency facilities like the big van embossed with your organization name so that people will know this is your van, not an ordinary van. It should be parked 10 to 15 minutes far from home. If it will be available 24 hours, it is good.” (IDI: Community leader – Ali Akbar Shah Site)*.

*“Water and soap should be available for cleaning. Every system should be placed in the van it should be like an ambulance ( equipped with facilities).” (IDI: Grandfather of deceased infant – Ali Akbar Shah Site)*.

### 6. Role of monetary support for death notifications within the community

Healthcare providers and community leaders suggested using local clinics to source community-level death alerts as they have wide range and network within the community. Further, most of the community leaders recommended providing monetary incentives for individuals who provide information to the sample collection teams to encourage timely reports of death alerts of children. Moreover, healthcare workers proposed monetary help to the parents and relatives in terms of sharing the cost of the funeral ceremony.

*“You should provide good monetary incentive for those who help in sampling. You should give 1000 PKR (3.6 USD) to those who need support.” (IDI: Community leader – Bhains Colony and Ali Akbar Shah Sites)*.

*“Community is poor so if your study can provide monetary benefits to the community would be beneficial for them.” (FGD: Healthcare Providers of Ali Akbar Shah Site)*.

### 7. Empathy with bereaved parents within the community - grief support

The healthcare professionals mentioned that parents experience indescribable grief and societal pressure upon death of their children or stillbirth. The healthcare providers and religious leaders highlighted that parents of deceased children frequently suffer from depression; therefore, they proposed incorporating grief counseling as a part of MITS process. Respondents recommended that parents should get grief counseling for at least three months after the death of a child or having a stillbirth:

*“If it is possible, you should go for at least for 3 months to meet the grieving family.” (IDI: Community leader – Ali Akbar Shah Site)*.

### 8. Potential community-based barriers to implementing MITS

Some healthcare providers shared that, within the community, parents and relatives might not allow any healthcare provider to touch their baby’s corpse as they believe to do so is disrespectful towards the corpse, and that they may therefore find the MITS procedure suspicious:

*“They will not allow touching a dead body. People will be suspicious of what you people are doing with the child. Even if the family agrees for the MITS sample collection than their relative and neighbors will not show reservation.” (FGD: Healthcare provider of Bhains Colony Site)*.

The participants felt that, from an emotional and religious perspective, families in the community would not accept the use of needles on the deceased, as they believe it could cause harm and may lead to disfigurement of the child, thereby enhancing their grief.

#### Community fear of medical procedure

The healthcare providers shared their concern that a fear of needles and medical procedures might present a barrier to sample collection in these communities.

*“This is very difficult to collect samples from chest any parent would not allow pricking in their deceased child.” (IDI: Grandmother of deceased infant - Bhains Colony Site)*.

*“The surgical procedure is difficult the people would be afraid of the name of the needle.” (FGD: Healthcare provider of Bhains Colony Site)*.

Additionally, healthcare providers revealed that it might be difficult to inform the community about the MITS procedure and consent process because of low rates of literacy and a lack of understanding of medical procedures. Community leaders said that most of the population has low education levels, which might be a main factor for refusing consent during MITS sample collection. Some people might also not be able to read religious rulings, nor would they understand medical processes like sampling with the needle. Thus, healthcare workers predicted that community education and comprehension would be one of the most significant obstacles to the implementation of the MITS project.

*“In our area, 80% of people are illiterate, they can cause problems. It is a difficult procedure to get a sample with a needle. If we make them understand (about MITS) they will agree.” (IDI: Community Leader - Bhains Colony Site)*.

#### Community concerns about MITS misconceptions

Both healthcare providers and parents identified negative comments and misinformation as challenges for MITS procedure: these misconceptions may create barriers to acceptance and understanding. Effective communication and education are essential in addressing community concerns and providing accurate information to parents. Without communication and education, relatives may find participation in MITS sampling negative, and families might stigmatize families. While it is important to counsel parents as the main decision-makers in the family, participants shared that it was essential to advocate with relatives in the wider community, as they may have reservations about the MITS procedure.

*“Relatives and others will not allow us to take samples in the van. Therefore, you should counsel parents and relatives to understand to get sample in the van.” (IDI: Parent of deceased child – Ali Akbar Shah Site)*.

*“Relatives’ reaction about MITS Sampling in van, people always gossip whether you do good or bad, it is quite usual. If the father agrees, you will get the sample without any problem.” (FGD: Healthcare provider of Bhains Colony Site)*.

## Discussion

This study is one of the first qualitative research of its kind to examine the practicality of performing minimally invasive tissue sampling (MITS) procedures in communities (locations outside of healthcare facilities) in a resource-constrained setting. It is a significant contribution because MITS is a useful tool for postmortem diagnosis, but it is typically conducted in hospitals or similar settings. By exploring the potential for MITS to be performed outside of these conventional settings, the study provides valuable insights into how the MITS procedure could be modified to address the needs of the community in various contexts. According to our study results, household elders and parents have the desire to understand the cause of their child’s death: this can bring closure to the family, which can be important for the grieving process. They also saw MITS as a less intrusive method that could bring benefits to the community in terms of saving future generations. It was emphasized that counseling for parents was essential to address emotional issues related to MITS acceptance. Other studies have also highlighted the importance of providing comprehensive information and emotional support to families through counseling [28, 29].

Muslim experts from Egypt and Syria have published legal views (referred to as Fatwas) stating that these religious edicts may be disregarded if there is a compelling cause to perform an autopsy, such as benefit to society or disease prevention [30]. A study by Gurley et al. have identified fear of physical deformity, cultural and religious objections as primary obstacles to the acceptance of MITS or biopsies in Muslim communities [28]. Feroz et al. in Pakistan found that acceptance of MITS was strongly influenced by religious perspectives, respect for the deceased, and minimal damage to the body, with the consent of guardians being mandatory when performing MITS even at the hospital level [20]. However, another study in Africa found that the majority of the Muslims would be willing to know the cause of death (78%) and accept MITS (70%) [30]. Similarly, in our study, the findings emphasize the role of religion and religious leaders in influencing the community’s acceptance of MITS sampling. Fatwa could play a significant role in shaping the community’s perception of MITS, with funeral workers and community leaders emphasizing that if Sharia permits it, then sampling should be allowed. This suggests that religious leaders may have a crucial role in shaping the community’s attitudes concern towards new medical procedures. The study also revealed varying perspectives on MITS among participants, with some parents concerned that the identified cause of death might affect their lives, particularly women who may face negative consequences. Additionally, parents of deceased children shared that knowing the cause of death could help parents overcome their self-blame and guilt for their children’s death. These findings suggest that MITS may have both positive and negative implications for families who have lost a child. Another study conducted in Pakistan also found that a major reason for accepting MITS was the desire to know the cause of death for their children [21]. Similarly, our study also found that parents were willing to give their consent for MITS to determine the cause of death for their child, and they were comfortable with the diagnosis being shared with them while maintaining confidentiality.

Therefore, maintaining confidentiality while disclosing the findings of MITS to parents, involving parents and family counseling to ensure effective MITS, community-level death alerts, and monetary incentives for individuals who provide information to sample collecting teams can be useful in obtaining death-related information. Incorporating community grief counseling into MITS can ensure that parents are aware of and understand the cause of death, and empowering bereaved parents through grief support can involve giving monetary help to share the cost of the funeral ceremony and grief counseling.

Our study identified that underserved communities have widespread fear and skepticism towards MITS due to a lack of awareness and misinformation about the procedure, as well as concerns about community conduct and rumors. One feasible way to address this issue is to implement a rumor surveillance system, as described by Islam et al. in Bangladesh, Mali, and Mozambique, which involves young volunteers, local stakeholders, community leaders, and study staff in identifying and tracking rumors about MITS in the neighborhood. They also addressed rumors through community meetings and education programs. Additionally, study participants suggested that community outreach and education programs could increase awareness and dispel myths about MITS [31]. It is crucial to conduct qualitative research that addresses cultural sensitivities, such as religious beliefs, practices, and concerns regarding infant mortality. Our study found that rumors about the child’s death and the involvement of MITS could hinder acceptance and consent.

**Fig. 2:**
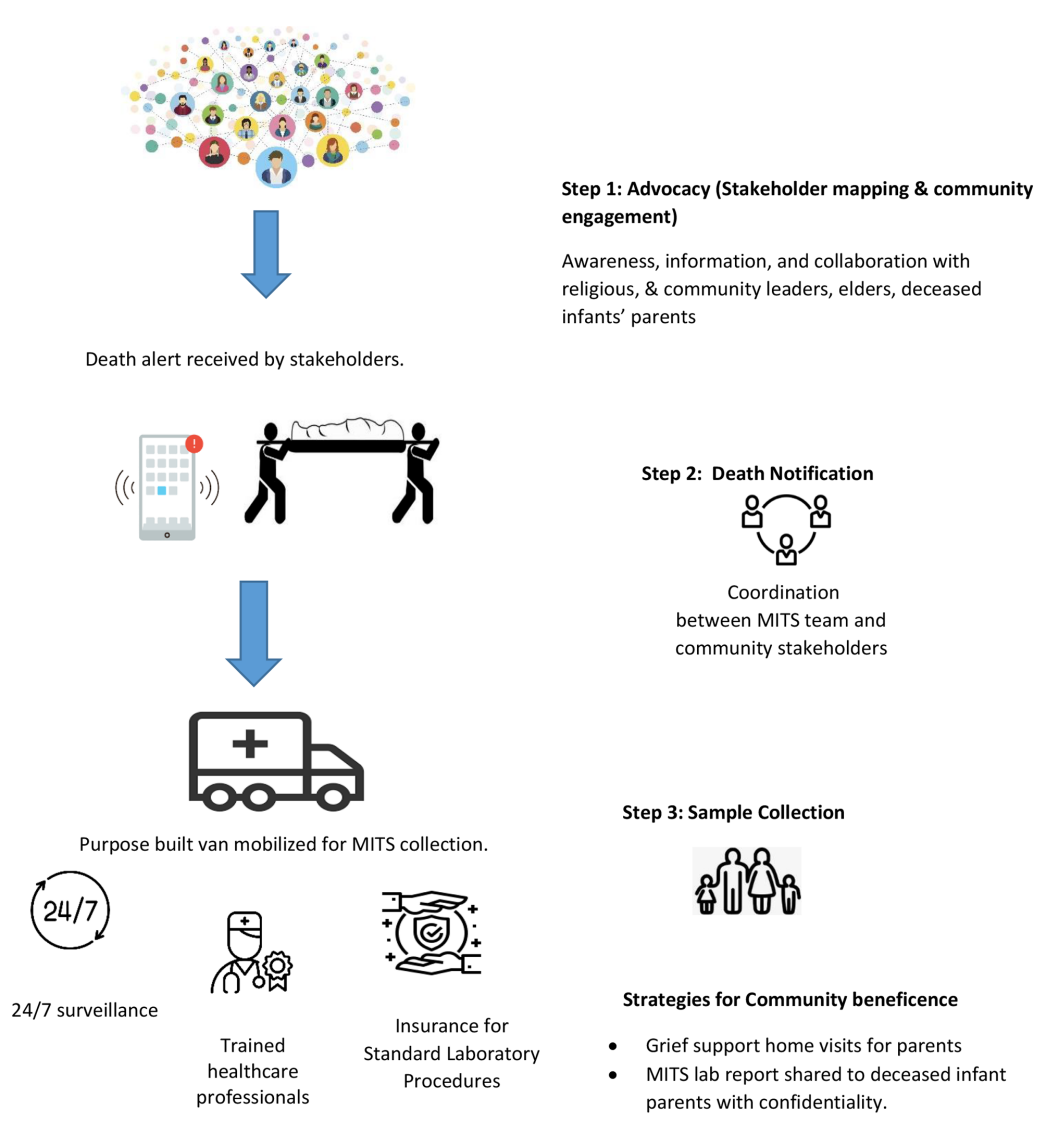
Derived Framework for Implementation of MITS at the Community Level

Lastly, our study aimed to investigate the feasibility of using a mobile van to conduct MITS in the community, near the home of the deceased child, to increase acceptance of the procedure. The community perceived sample collection van to be similar just like an ambulance, maintaining an infection control environment inside. Therefore, our study suggests that community-based sample collection through a mobile van could improve acceptability among community members. The study implementation framework is derived from the formative phase of mortality studies conducted within the community which describes the process of MITS surveillance (Fig. 2).

The findings from this qualitative study proposed results derived framework on how to implement MITS procedure within the resource constrain community setting of LMICs. This framework comprises of three major steps described below.

1. Advocacy: Stakeholder mapping and ongoing community engagement.
2. Process of receiving death notifications from within the community.
3. Standard operating procedures for MITS sample collection using specially designed van and trained medical staff followed by grief support for bereaved parents through study psychologist.

The major limitation of this study is that the acceptance and feasibility of MITS was explored with community members in a hypothetical manner. Further, findings based on the opinions of other communities in Pakistan may differ, therefore, further participatory research on a larger scale is necessary to achieve generalizability within the population. Lastly, the lack of awareness of MITS among community members may have impacted the study’s results and affected its validity.

## Conclusion

This study provides valuable insights into the community’s perceptions and beliefs regarding the implementation of minimally invasive tissue sampling (MITS) procedures in Pakistan. The results suggest that conducting MITS sample collection in the community can be an acceptable approach to determine the cause of death in newborns and stillbirths, if the body of the deceased is treated with respect and buried promptly. The study’s findings highlight the need for a community-based approach to effectively implement and integrate MITS into the current healthcare system in Pakistan, including the use of a dedicated sample collection van and grief support related counseling. These insights can inform the development of initiatives that would be helpful for improving MITS acceptance and implementation in underserved communities.

### Electronic supplementary material

Below is the link to the electronic supplementary material.

Supplementary Material 1

## Data Availability

The datasets used and/or analyzed during the current study are available from the corresponding author on reasonable request.

## Abbreviations

AAG: Ali Akbar Shah
AKU: Aga Khan University
ANC: Antenatal care
CDA: Complete diagnostic autopsy
CHWs: Community Health Workers
CoD: Cause of death
FGDs: Focus group discussions
HCP: Healthcare Providers
HDSS: Health demographic surveillance system
ID: Identification
IDI: In-depth interviews
LHWs: Lady Health Workers
LMICs: Low middle-income countries
MITS: Minimally invasive tissue sampling
SIDS: Sudden Infant Death Syndrome
UCCSO: Union Council Communication Support Officers
VA: Verbal autopsy
