# Supplementary material for: Postmortem minimally invasive tissue sampling in communities: exploring perceptions of families, funeral workers, religious and community leaders and healthcare providers from Pakistan
Source: BMC Health Serv Res. 2023 Dec 13;23:1402. doi: 10.1186/s12913-023-10438-2 (PMC10720140; doi:10.1186/s12913-023-10438-2)
Supplement: Supplementary file 1 — Supplementary Material 1 [file 12913_2023_10438_MOESM1_ESM.docx]

**Qualitative In-Depth and FGD Interview Guide**

1. What are the major illnesses due to which infant death occur in your area?

Probe:

- Due to seasonal illness

1. In your opinion, where do people go to seek treatment during a child emergency?
2. It would happen Is it possible that you are not able to don’t identify the reason for infant death? Is it common in your community?

Probe:

- In such cases, what are the opinions and feelings of the family of the deceased about trying to ascertain the diseases which cause the death?

1. In your opinion what are the causes (pregnancy related) which result in neonatal death?

**Probes:**

- Common beliefs and practices around early pregnancy loss (death occur at household/hospital)

**Script 1: MITs description**

1. Is it possible to take MITs samples of deceased infants? Will this be acceptable among people in your community?

**Probe:**

- Will people allow us- why? (hospital, household, ambulance)
- If no, then explore what were the reasons behind refusal to this

1. Where do you think is the ideal place to collect the MITS sample from the child?
2. What do you think about sample collection in an ambulance?

Probe:

- A proper equipped ambulance to collect NP/MITs sample which consist of seeking arrangement for staff, arrangement for deceased body, water and table equipped and fully air conditioned ambulance

1. What are the concerns for the use of MITS procedure in a deceased neonate?

**Probes:**

- Concerns related to deceased body
- Religious beliefs

1. What are the perceived facilitators for the implementation of MITS procedure?

**Probes:**

- Community buy-in
- Religious buy-in
- Stake holder buy-in (Key Informants)
- clear information about MITs procedure

1. What advice would you give to such a parent who are unsure about allowing the MITs to be taken?
2. Is this religiously acceptable to obtain a MITs sample from a deceased infant?
3. What are the advantages for the implementation of MITS procedure?

**Probe:**

- To understand the cause of death in deceased neonate
- To prevent further deaths

These are all the questions I had for you today. Do you have any questions for me?
